# Psychometric Evaluation of the Naglieri Nonverbal Ability Test (NNAT-I) in Turkish Middle School Students Aged 10–14 Years

**DOI:** 10.3390/jintelligence14090209

**Published:** 2026-09-01

**Authors:** Sevinç Zeynep Kavruk, Ahmet Bildiren

**Affiliations:** 1 Department of Child Development, Faculty of Health Sciences, Aydın Adnan Menderes University, Aydın 09100, Türkiye; 2 Department of Special Education, Faculty of Education, Aydın Adnan Menderes University, Aydın 09100, Türkiye; abildiren@adu.edu.tr

**Keywords:** nonverbal intelligence, NNAT-I, psychometric evaluation, cognitive assessment, Rasch analysis

## Abstract

The Naglieri Nonverbal Ability Test–Individual Form (NNAT-I) is widely used to assess nonverbal reasoning while minimizing language demands, yet evidence regarding its psychometric properties in Turkish middle school students is limited. This study examined the reliability, convergent validity, internal structure, item functioning, and differential item functioning (DIF) of the NNAT-I in 1130 students aged 10–14 years attending schools across socioeconomic development strata in Aydın, Türkiye. Internal consistency, test–retest and alternate-form reliability, convergent validity, confirmatory factor analysis (CFA), Rasch analyses, multiple-group Rasch DIF analyses, and linear mixed-effects modeling were conducted. The NNAT-I demonstrated high internal consistency (Cronbach’s α = 0.93; McDonald’s ω = 0.94), strong test–retest and alternate-form reliability, and strong correlations with the Test of Nonverbal Intelligence–Third Edition (TONI-3) and Raven’s Standard Progressive Matrices (RSPM). CFA provided qualified rather than unequivocal evidence for a predominantly unidimensional structure, with model fit varying according to item response distributions and the treatment of structurally nonadministered responses. Rasch analyses of the calibrated items supported an ordered hierarchy of item difficulty, with all items showing Infit values within the prespecified descriptive reference range. After adjustment for multiple comparisons, DIF analyses identified significant gender-related DIF for only one item, while no items showed significant age-related DIF. Linear mixed-effects modeling showed that grade level and gender were significantly associated with NNAT-I scores, whereas chronological age was not independently associated with performance. These findings provide evidence regarding the reliability, convergent validity, internal structure, and item functioning of the NNAT-I in this Turkish middle-school sample, while also indicating that conclusions regarding unidimensionality should remain qualified. The findings underscore the importance of local psychometric evaluation before applying nonverbal measures in new educational and cultural contexts.

## 1. Introduction

Intelligence assessment directly informs educational placement, clinical diagnoses, and gifted identification. However, many established instruments draw heavily on verbal comprehension, expressive language, and prior academic exposure—factors that can confound measured cognitive capacity (Roid & Barram, 2004; Wechsler, 2014). To address these linguistic and background confounds, nonverbal measures assess reasoning through visual stimuli, minimizing language demands during administration (Brown, 2003; Friedlander Moore et al., 2017; Hammill & Pearson, 2017; Naglieri, 2003).

Nonverbal tests do not measure a distinct construct of intelligence; rather, they evaluate general reasoning through nonverbal response formats. By emphasizing fluid reasoning, visual–spatial pattern recognition, abstract problem solving, and inductive logic, these instruments reduce reliance on literacy and spoken language. Consequently, they are widely applied in culturally and linguistically diverse settings to promote equitable cognitive assessment (Brown, 2003; Naglieri, 2003). Still, minimizing language demands does not render an assessment entirely immune to cultural or environmental context.

The Naglieri Nonverbal Ability Test (NNAT) is prominently featured in efforts to mitigate linguistic bias in gifted education (Naglieri, 2003), yet it remains subject to ongoing debate. Meta-analytic findings indicate satisfactory reliability and moderate criterion validity, particularly for academic achievement (Lee et al., 2021). However, nonverbal testing alone does not eliminate identification disparities, as outcomes may remain associated with demographic and educational factors (Carman et al., 2020; Hodges et al., 2018). Recent studies similarly report demographic differences and emphasize that NNAT scores should be interpreted within broader assessment protocols rather than as standalone measures (Alodat, 2026; Selvamenan et al., 2024; Toornstra et al., 2020). Much of the available psychometric evidence for the NNAT, however, originates from North American samples, leaving its performance in other educational and cultural contexts less well established. This limitation is important because psychometric properties demonstrated in one population cannot be assumed to generalize to another.

The broader literature also indicates that nonverbal reasoning is associated with developmental, educational, and environmental factors and may show distinct patterns across different populations (Georgiou, 2023; Goodman et al., 2022, 2023; Madhushanthi et al., 2020; Tamayo Martinez et al., 2022; Zakharov et al., 2020). Clinical and developmental studies further illustrate the value of assessing nonverbal reasoning while also showing that nonverbal scores alone do not capture the full range of cognitive and academic functioning (Grantham et al., 2022; Onnivello et al., 2022; Operto et al., 2020; Silleresi et al., 2020).

Recent psychometric studies of nonverbal ability measures have emphasized the importance of evaluating reliability, internal structure, convergent validity, and measurement comparability within the populations in which these instruments are used. In Türkiye, the Bildiren Nonverbal Ability Test (BNV), developed for children aged 4–13 years, demonstrated high internal consistency and test–retest reliability in a large national sample and showed substantial correlations with the NNAT, TONI-3, and Colored Progressive Matrices (Bildiren et al., 2021). Research on other nonverbal measures has also incorporated latent-variable approaches and tests of measurement invariance, highlighting that satisfactory reliability alone does not establish equivalent measurement across demographic groups (Benson et al., 2020). Turkish research on the CTONI-2 has further demonstrated the value of combining internal-consistency estimates, confirmatory factor analysis, test–retest reliability, and convergent validity evidence when evaluating nonverbal measures in new linguistic and educational settings (Ozdemir et al., 2026). This recent literature highlights the value of evaluating nonverbal measures using multiple sources of psychometric evidence beyond conventional reliability coefficients.

Despite these developments in the psychometric evaluation of nonverbal measures in Türkiye, evidence specific to the NNAT-I remains limited. Reliability and validity evidence is available for Turkish children aged 5–9 years (Kavruk & Bildiren, 2022), but corresponding evidence for middle school students aged 10–14 years is lacking. This represents an important gap because psychometric properties established in younger children cannot be assumed to extend to early adolescence, when abstract reasoning demands and educational differentiation increase. Moreover, the present study extends previous Turkish research by incorporating Rasch analysis, differential item functioning, and multilevel modeling alongside classical psychometric approaches.

The present study was guided by complementary principles from classical test theory and Rasch measurement. From a classical test theory perspective, a psychometrically sound measure of nonverbal reasoning is expected to demonstrate adequate reliability and meaningful associations with established measures of the same or closely related constructs. From a Rasch measurement perspective, items measuring a common latent ability are expected to form an ordered difficulty continuum and generally demonstrate acceptable model fit. In addition, given the intended reduction in linguistic and cultural demands in nonverbal assessment, examining whether individual items function differently across demographic groups is important for evaluating the psychometric performance of the instrument across diverse student groups.

Although recent studies have expanded the psychometric evidence available for nonverbal ability assessment in Türkiye (Bildiren et al., 2021; Bildiren et al., 2025; Kavruk & Bildiren, 2022; Ozdemir et al., 2026), evidence specific to the NNAT-I in middle school students remains limited. Existing Turkish research on the NNAT has primarily focused on younger children, leaving its psychometric performance during early adolescence insufficiently examined. Accordingly, the present study evaluates the NNAT-I in Turkish middle school students aged 10–14 years using both classical and modern psychometric approaches. Specifically, we examine reliability, evidence of convergent validity, item difficulty and discrimination, Rasch model fit, differential item functioning (DIF) across gender and age groups, and multilevel demographic associations. Based on this theoretical framework and previous psychometric evidence, the following hypotheses were formulated: (H1) the NNAT-I would demonstrate adequate internal consistency, test–retest reliability, and alternate-form reliability; (H2) NNAT-I scores would be meaningfully and positively associated with established measures of nonverbal reasoning, specifically the TONI-3 and Raven’s Standard Progressive Matrices; (H3) the calibrated NNAT-I items would exhibit an ordered difficulty hierarchy and generally acceptable fit to the Rasch model; and (H4) the calibrated items would show limited DIF across gender and age groups. Given the mixed evidence regarding demographic differences in nonverbal ability performance, associations of grade level, chronological age, and gender with NNAT-I scores were examined exploratorily rather than specified as directional hypotheses. In this way, the study provides age-specific and item-level psychometric evidence for the NNAT-I in an age group not represented in previous Turkish research and contributes to the broader psychometric literature on nonverbal ability assessment across educational and cultural contexts.

## 2. Materials and Methods

### 2.1. Participants

For the sampling framework, the most recent official enrollment statistics available at the time of the study were used. These statistics indicated that 57,213 students were enrolled in middle schools across Aydın Province during the 2024–2025 academic year (MoNE, 2025). Sample selection was based on the Socio-Economic Development Ranking Research (SEGE) developed by the Ministry of Industry and Technology of the Republic of Türkiye. The SEGE index ranks provinces and districts according to their level of socioeconomic development using indicators from multiple domains, including education, health, employment, financial capacity, and infrastructure (MoIT, 2022). According to the 2022 SEGE classification, the districts of Aydın Province represented four socioeconomic development levels.

A stratified cluster sampling design was used, with SEGE development level serving as the stratification variable and schools constituting the sampling clusters. Schools were randomly selected within each SEGE stratum. Because districts in the first SEGE stratum contained a substantially larger middle-school student population, three schools were selected from this stratum, whereas one school was selected from each of the remaining three strata. The resulting sample comprised six schools across four districts. School cluster sizes ranged from 103 to 256 students. The first SEGE stratum included three schools and 503 participants, whereas the second, third, and fourth strata were each represented by one school, contributing 231, 256, and 140 participants, respectively.

Within the selected schools, all eligible students enrolled in Grades 5 through 8 were invited to participate. A total of 1200 eligible students were invited, of whom 1130 participated, corresponding to a participation rate of 94.2%. The final analytic sample therefore consisted of 1130 students aged between 10 years 0 months and 14 years 6 months. The demographic characteristics of the participants according to gender, age, grade level, and socioeconomic development category are presented in Table 1.

### 2.2. Measurements

The Naglieri Nonverbal Ability Test–I (NNAT-I), developed by Naglieri (2003), is a nonverbal assessment designed to minimize language demands while evaluating general cognitive ability. The instrument consists of 72 matrix-based items requiring individuals to identify relationships among visual patterns and complete missing components. Responses are scored dichotomously, with correct answers coded as 1 and incorrect answers coded as 0. The original standardization study of NNAT-I was conducted in the United States with a sample of 1585 individuals between 5 and 17 years of age. Findings from the original study demonstrated strong psychometric properties, including a Cronbach’s alpha coefficient of 0.91 and a test–retest reliability coefficient of 0.71. Evidence for construct validity was supported through substantial correlations with other nonverbal cognitive measures. Specifically, correlations were reported as 0.78 with the Raven’s Standard Progressive Matrices (RSPM), 0.63 with the Test of Nonverbal Intelligence–Third Edition (TONI-3), and between 0.31 and 0.65 with the Wechsler Intelligence Scale for Children–Fourth Edition (WISC-IV) (Naglieri, 2003). In the present study, NNAT-I raw scores were used to examine convergent validity through their associations with other measures of nonverbal reasoning.

The Test of Nonverbal Intelligence–Third Edition (TONI-3), developed by Brown et al. (1997), is a nonverbal measure designed to assess general intellectual functioning independent of language proficiency, motor skills, and cultural background. The test can be administered to individuals aged 6 to 89 years and primarily evaluates abstract reasoning through visually presented problem-solving tasks. The Turkish norming and standardization study of TONI-3 was carried out by Korkmaz et al. (2018) with a sample of 631 children aged between 6 and 11 years. In that study, KR-20 internal consistency coefficients ranged from 0.86 to 0.95 for Form A and from 0.90 to 0.93 for Form B. Additionally, strong positive correlations were found between TONI-3 and the Raven’s Standard Progressive Matrices, with coefficients of 0.79 for Form A and 0.82 for Form B, supporting the criterion validity of the instrument.

The Raven’s Standard Progressive Matrices (RSPM), introduced by Raven (1983), is a widely used nonverbal assessment instrument aimed at measuring general reasoning ability. The scale includes 60 items divided into five sets (A–E), each composed of 12 progressively difficult problems. Participants are required to identify the missing part of a geometric pattern by selecting the correct option from a set of alternatives. Item difficulty increases both within and across sets as the visual relationships become more complex. The Turkish adaptation and standardization study of the RSPM was conducted by Şahin and Düzen (1994) with a sample of 2277 children aged 6–15 years. The split-half reliability coefficient for the overall scale was reported as 0.91. Moreover, correlations among subtests ranged from 0.58 to 0.95, indicating satisfactory construct validity and internal consistency. In the present study, raw RSPM scores were used to examine convergent validity through their association with NNAT-I scores.

### 2.3. Procedure

Prior to data collection, ethical approval for the study was obtained from the Aydın Adnan Menderes University Educational Research Ethics Committee (Decision No. 2026/VII, dated 30 January 2026). Following ethical approval, official authorization to conduct the study in schools was granted by the Ministry of National Education. School administrations were subsequently contacted, and assessment schedules were organized in a manner that would not interfere with routine educational activities in the selected schools. Written informed consent was obtained from the parents or legal guardians of all participants before their participation in the study. Data collection was conducted between February and June 2026.

Before the administration process, written informed consent was obtained from the parents or legal guardians of all participants. NNAT-I Form A was used as the primary form for the main assessment. The test was administered individually to each participant by the first author, a specialist in child development, in accordance with the standardized individual administration procedures specified in the 2003 NNAT-I manual. Testing was conducted in an appropriate setting with distractions minimized. Before proceeding to the test items, the standardized instructions and practice items were presented individually to ensure that each participant understood the task requirements. Following the practice items, each participant completed the test individually in accordance with the standardized administration and discontinuation procedures described in the manual. The administration process lasted approximately 30 min per participant.

Additional procedures were conducted to evaluate the psychometric properties of the NNAT-I. Because repeated administrations and additional measures could not feasibly be administered to the entire sample within the school-based data collection process, subsamples were randomly selected from the main study sample across participating schools. Test–retest reliability was evaluated in 105 participants who completed Form A twice, with a four-week interval between administrations. Alternate-form reliability was examined in 107 participants who completed Form A followed by Form B one week later; the order of administration was fixed and was not counterbalanced. Intraclass correlation coefficients (ICCs) with 95% confidence intervals were calculated to quantify agreement between repeated administrations and between the two forms. Convergent validity evidence was examined through associations between NNAT-I scores and two established measures of nonverbal reasoning: the Test of Nonverbal Intelligence–Third Edition (TONI-3) in 171 participants and Raven’s Standard Progressive Matrices (RSPM) in 98 participants. Some participants contributed to more than one of these analyses. A power analysis indicated that a minimum sample size of 85 was required to detect a moderate correlation (*r* = 0.30) with 80% power at α = 0.05 (two-tailed). Accordingly, all subsamples exceeded this requirement, and the smallest subsample (*n* = 98) provided 80% power to detect correlations of approximately *r* = 0.28 or greater.

### 2.4. Statistical Analysis

All statistical analyses were conducted using IBM SPSS Statistics version 25.0, JASP version 0.18.3.0, and R version 4.5.3. Descriptive statistics, including frequencies, percentages, means, standard deviations, minimum values, and maximum values, were calculated for NNAT-I scores according to gender, age group, grade level, and socioeconomic development level (SEGE).

The internal consistency of the NNAT-I was evaluated using Cronbach’s alpha and McDonald’s omega coefficients in JASP. Because the NNAT-I items were dichotomously scored (0 = incorrect, 1 = correct), Cronbach’s alpha was interpreted as equivalent to the Kuder–Richardson Formula 20 (KR-20) coefficient. McDonald’s omega was estimated using the principal factor analysis (PFA) approach implemented in JASP.

Construct validity was further examined using confirmatory factor analysis (CFA) to evaluate the factorial structure of the NNAT-I. Because the items were dichotomously scored (0/1), CFA was conducted using the weighted least squares mean- and variance-adjusted (WLSMV) estimator, treating the items as ordered categorical variables. A one-factor model was specified in accordance with the intended measurement of a common nonverbal reasoning ability. Consistent with the Rasch calibration, the primary CFA was conducted using Items 10–72, as Items 1–9 showed pronounced ceiling effects and insufficient response variability. Model fit was evaluated using the comparative fit index (CFI), Tucker–Lewis index (TLI), root mean square error of approximation (RMSEA), and standardized root mean square residual (SRMR), with scaled CFI, TLI, and RMSEA values additionally reported for the WLSMV estimation. Because extreme item response distributions may affect categorical CFA estimates, a sensitivity analysis was additionally conducted including only items for which both response categories represented at least 5% of observations (Items 21–65). To examine the influence of structural nonadministration associated with the discontinue rule, an additional CFA sensitivity analysis was conducted using the reconstructed response dataset employed in the Rasch sensitivity analysis, in which the four observed incorrect responses triggering discontinuation were retained as incorrect and subsequent nonadministered items were treated as missing. This analysis was first attempted with Items 10–72 and, because that model did not converge, was subsequently estimated using the Items 21–65 sensitivity subset. CFA analyses were performed in R (version 4.5.2) using the *lavaan* package (version 0.7-2). Standardized factor loadings and item thresholds were also obtained from the primary one-factor model and are reported in the Supplementary Materials.

In addition to classical test theory analyses, item-level psychometric analyses were conducted to provide complementary evidence regarding the internal structure and measurement properties of the NNAT-I. Item difficulty indices and corrected item–total correlation coefficients were calculated to evaluate item characteristics and discrimination. Furthermore, a one-parameter Rasch item response theory (IRT) model was fitted to estimate item difficulty parameters and examine model fit. Rasch item statistics, including item difficulty estimates, infit mean square (Infit MnSq) and outfit mean square (Outfit MnSq) statistics, were examined to evaluate the extent to which individual items conformed to model expectations. Infit and Outfit MnSq values between 0.70 and 1.30 were used as descriptive reference ranges for evaluating item fit rather than as absolute criteria for determining satisfactory model fit. Item-fit statistics were interpreted in conjunction with item difficulty, response distributions, the number of participants for whom each item was actually administered, and the results of the sensitivity analysis. Rasch model estimation was performed in R using the TAM package (version 4.3-25).

Prior to Rasch calibration, item response distributions were examined. Items 1–9 exhibited pronounced ceiling effects and very limited response variability, which did not permit stable and informative estimation of their item difficulty parameters. These items were therefore excluded from the Rasch calibration. Consequently, the Rasch-based item-level results reported in this study, including the DIF analyses, pertain to Items 10–72 and should not be interpreted as representing the Rasch-based item functioning of Items 1–9. Because the NNAT-I administration was discontinued after four consecutive incorrect responses, items following the discontinue point were not administered. In the original scoring dataset, these nonadministered items were coded as 0 in accordance with the raw-score scoring procedure. The zero-coded model was retained as the primary Rasch analysis because it corresponded to the NNAT-I raw-score scoring convention and the scoring structure used for the primary classical analyses. However, this scoring convention does not distinguish observed incorrect responses from structurally nonadministered items and therefore does not necessarily represent the preferred treatment of responses for item-response calibration. To evaluate the potential influence of this coding convention on Rasch calibration, a sensitivity analysis was conducted. The four observed incorrect responses that triggered discontinuation were retained as incorrect (0), whereas all subsequent nonadministered items were recoded as missing. This reconstructed-response analysis was treated as a complementary psychometric calibration to evaluate the extent to which the Rasch results depended on the raw-score coding convention. The same one-parameter Rasch model was then re-estimated using Items 10–72. Item-difficulty estimates, Infit and Outfit statistics, and EAP reliability from the sensitivity analysis were compared with those obtained from the primary Rasch analysis. The number of participants for whom each item was actually administered was also calculated.

Local independence was examined using Yen’s Q3 residual correlations following Rasch model estimation. Because raw Q3 values may have a non-zero average under Rasch models, adjusted Q3 (aQ3) values, obtained by centering pairwise Q3 statistics around their overall mean, were also examined. The magnitude and distribution of aQ3 values were used to identify item pairs showing comparatively elevated residual associations. Local dependence was evaluated for both the primary and sensitivity Rasch models. Because the number of jointly administered responses decreased for later item pairs in the sensitivity analysis, the number of observations contributing to each pairwise residual correlation was also considered when interpreting elevated aQ3 values. Residual dependence analyses were conducted using the TAM package (version 4.3-25). To investigate whether NNAT-I items functioned similarly across gender and age groups, differential item functioning (DIF) analyses were conducted within a multiple-group Rasch IRT framework. Separate multiple-group Rasch models were estimated for gender (female vs. male) and age (10, 11, 12, 13, and 14 years). In the baseline models, item difficulty parameters were constrained to be equal across groups, whereas latent means and variances were freely estimated. DIF was evaluated using likelihood-ratio tests under the drop-one-item procedure, whereby equality constraints on the item difficulty parameter were sequentially released for each item while the remaining items served as anchor items. Because the Rasch model assumes equal item discrimination across items, only item difficulty parameters were evaluated. To control for multiple testing, *p* values were adjusted using the Benjamini–Hochberg false discovery rate (FDR) procedure. For items demonstrating nominal evidence of DIF, group-specific item difficulty estimates were compared and difficulty contrasts (logits) were examined to aid interpretation. Multiple-group DIF analyses were performed in R using the mirt package (version 1.46.1). As a robustness check, the gender- and age-based DIF analyses were repeated using the sensitivity dataset, in which post-discontinuation nonadministered responses were treated as missing. In addition, a purified-anchor sensitivity analysis was conducted to assess the potential influence of anchor contamination on the gender DIF finding. Items showing nominal evidence of gender DIF (*p* < .05) in the initial sensitivity analysis were excluded from the anchor set, leaving 54 items as purified anchors. Item 26 was then retested for gender DIF using this purified anchor set, and its group-specific difficulty estimates and absolute difficulty contrast were examined.

Test–retest reliability and alternate-form reliability were evaluated using Pearson correlation coefficients and intraclass correlation coefficients (ICCs), estimated with a two-way mixed-effects model for absolute agreement and single measurements. Standard errors of measurement (SEM) were calculated from the corresponding ICC estimates. These analyses were performed using IBM SPSS Statistics version 25.0.

Evidence of convergent validity was evaluated using Pearson correlation coefficients between NNAT-I scores and scores on the Test of Nonverbal Intelligence–Third Edition (TONI-3) and Raven’s Standard Progressive Matrices (RSPM). In addition, age-adjusted partial correlations controlling for chronological age were computed. The TONI-3 was administered in accordance with the standardized administration procedures specified in the test manual, including the prescribed discontinuation rule, and participants’ performance was evaluated using raw scores. These analyses were conducted in IBM SPSS Statistics version 25.0.

Because students were nested within schools as a result of the stratified cluster sampling design, a null random-intercept model with school as a random effect was first estimated to quantify between-school variance and calculate the intraclass correlation coefficient (ICC). A linear mixed-effects model was then fitted with school specified as a random intercept to examine the associations of age, grade level, and gender with NNAT-I total scores while accounting for the clustered data structure. Age, grade level, and gender were entered as fixed effects, with Grade 8 and male students serving as the reference categories for grade level and gender, respectively. The null model yielded a school-level ICC of 0.015, indicating limited between-school dependence. Given the small magnitude of the observed school-level variance, the Rasch and DIF analyses were conducted at the individual level rather than within a multilevel IRT framework.

SEGE was defined at the district level, with each school belonging to a single SEGE category. To examine its potential contextual association with NNAT-I performance, SEGE was additionally entered as a school-level fixed predictor in a sensitivity mixed-effects model. Because the study included only six school clusters and three of the four SEGE strata were represented by a single school, the SEGE effect was interpreted cautiously given the limited independent information available at the school level.

Model assumptions were evaluated using residual diagnostics. Residual-versus-fitted plots were examined to assess homoscedasticity, Q–Q plots were inspected to evaluate the approximate normality of residuals, and standardized residuals were examined for extreme observations. Given the strong association between chronological age and grade level, their relationship was further examined using Pearson and Spearman correlations, and collinearity was assessed using variance inflation factor (VIF) and tolerance statistics. To evaluate the stability of their associations with NNAT-I performance, additional sensitivity mixed-effects models were estimated with (a) age as the sole developmental predictor, (b) grade level as the sole developmental predictor, and (c) age and grade level entered simultaneously. Gender was retained as a fixed effect and school as a random intercept in these sensitivity models. These analyses were used to examine how the estimated associations of age and grade changed when the two overlapping predictors were modeled separately and jointly.

Fixed-effect estimates are reported as regression coefficients (B), standard errors (SE), t- or F-statistics, 95% confidence intervals (CIs), and *p* values. Model fit was evaluated using the −2 Restricted Log Likelihood, Akaike Information Criterion (AIC), and Bayesian Information Criterion (BIC). The primary multilevel analyses were performed using IBM SPSS Statistics version 25.0. The additional sensitivity analyses were conducted in R using the lme4 and lmerTest packages.

## 3. Results

Descriptive statistics for NNAT-I scores across demographic variables are presented in Table 2.

Table 2 presents the descriptive statistics for NNAT-I scores according to gender, age group, grade level, and socioeconomic development level (SEGE). Mean NNAT-I scores were higher in older age groups and higher grade levels. Scores ranged from a mean of 36.71 (SD = 8.78) in the 10-year-old group to 44.57 (SD = 9.40) in the 14-year-old group. Similarly, mean scores ranged from 35.99 (SD = 8.78) in Grade 5 to 44.38 (SD = 9.39) in Grade 8. In contrast, mean NNAT-I scores were descriptively similar across SEGE strata.

Table 3 presents the McDonald’s omega and Cronbach’s alpha coefficients for the NNAT-I across gender, age groups, grade levels, and socioeconomic development levels (SEGE). Reliability coefficients were consistently high across the examined groups, indicating strong internal consistency of the test. Cronbach’s alpha coefficients ranged from 0.92 to 0.94 across all demographic groups and were 0.93 for both female and male students. McDonald’s omega coefficients ranged from 0.92 to 0.94 across the groups for which they could be estimated; omega estimates for the female, male, and 10-year-old groups could not be obtained because of computational convergence problems associated with perfectly correlated items. Overall, reliability estimates were consistently high across the examined demographic groups.

Table 4 presents the results of the confirmatory factor analyses. The primary one-factor CFA based on Items 10–72 provided mixed evidence regarding model fit. Although the scaled RMSEA was low (0.039), the scaled CFI (0.850), scaled TLI (0.845), and SRMR (0.169) indicated inadequate fit according to commonly used criteria. Standardized factor loadings ranged from 0.070 to 0.862 (see Supplementary Table S1 for item-level CFA parameters). Because several items exhibited extreme response distributions, a sensitivity analysis was conducted using Items 21–65, for which both response categories represented at least 5% of observations. Model fit improved substantially in this analysis (scaled CFI = 0.951, scaled TLI = 0.948, scaled RMSEA = 0.041, SRMR = 0.097), although the elevated SRMR indicated some residual misfit. To examine the potential influence of structural nonadministration associated with the discontinue rule, an additional CFA was attempted using the reconstructed response dataset employed in the Rasch sensitivity analysis, in which the four observed incorrect responses triggering discontinuation were retained as incorrect and subsequent nonadministered items were treated as missing. The model including Items 10–72 did not converge because of sparse pairwise coverage and empty bivariate response cells. The same structural-missingness approach was therefore applied to the prespecified Items 21–65 sensitivity subset. This model converged and yielded mixed fit (scaled CFI = 0.897, scaled TLI = 0.892, scaled RMSEA = 0.017, SRMR = 0.108). Standardized factor loadings ranged from 0.017 to 0.628, with the weakest loadings occurring among some of the later items, for which substantially fewer administered responses were available. Taken together, the CFA results provide qualified rather than unequivocal evidence for a predominantly unidimensional structure and indicate that conclusions regarding internal structure are sensitive both to extreme response distributions and to the structural nonadministration produced by the discontinue rule.

Table 5 presents a summary of the classical item statistics and the primary and sensitivity Rasch analyses for the NNAT-I. Item difficulty indices indicated that the test included items ranging from very easy to very difficult. Corrected item–total correlations ranged from −0.005 to 0.643, with 11 items showing coefficients below 0.20, including one slightly negative coefficient (Item 7, *r* = −0.005). Low discrimination coefficients were concentrated primarily among items with extreme response distributions. Items 3, 4, and 7, for example, were answered correctly by more than 99.9% of participants, indicating very limited response variability. Among the items included in the Rasch calibration, corrected item–total correlations below 0.20 were observed for Items 10–15 and 19, which were among the easier items, and for Item 72, which was at the difficult end of the test. These items nevertheless generally showed Infit values close to 1.00, indicating that low classical item discrimination was not consistently accompanied by Rasch Infit misfit.

Because Items 1–9 exhibited pronounced ceiling effects and insufficient response variability, they were excluded from the Rasch calibration; therefore, the Rasch results reported below pertain to Items 10–72. In the primary Rasch analysis, item difficulty estimates ranged from −7.89 to 6.04 logits. All 63 items demonstrated Infit mean-square values within the prespecified descriptive reference range of 0.70–1.30 (0.76–1.23), and EAP reliability was 0.941. However, because the raw-score scoring convention coded items not administered following the discontinue point as incorrect (0), the potential influence of this scoring convention on item-response calibration was examined through a sensitivity analysis. In this analysis, the four observed incorrect responses that triggered discontinuation were retained as incorrect, whereas subsequent nonadministered items were treated as missing.

The sensitivity analysis yielded item difficulty estimates ranging from −7.03 to 3.28 logits. The relative ordering of item difficulties was highly consistent with the primary analysis (*r* = 0.991), although absolute difficulty estimates changed, particularly for later items with fewer actual responses (mean absolute difference = 0.90 logits, median = 0.74, maximum = 2.83). All 63 items remained within the 0.70–1.30 Infit range, indicating that the Infit-based conclusions were robust to the alternative treatment of nonadministered responses. In contrast, the number of items within the 0.70–1.30 Outfit range increased from 17 of 63 in the primary analysis to 56 of 63 in the sensitivity analysis. Item 19 provided a particularly notable example, with a markedly elevated primary Outfit MnSq of 6.51 despite an Infit MnSq of 0.98; in the sensitivity analysis, its Outfit decreased substantially to 1.55, illustrating the sensitivity of Outfit statistics to the treatment of post-discontinuation responses. EAP reliability decreased from 0.941 to 0.855 but remained high. Overall, the relative item-difficulty hierarchy and Infit results were highly stable, whereas absolute difficulty estimates for later items and Outfit statistics were more sensitive to the treatment of nonadministered responses. Detailed primary and sensitivity Rasch item statistics, together with the number of participants for whom each item was actually administered, are provided in Supplementary Table S2.

**Figure 1:**
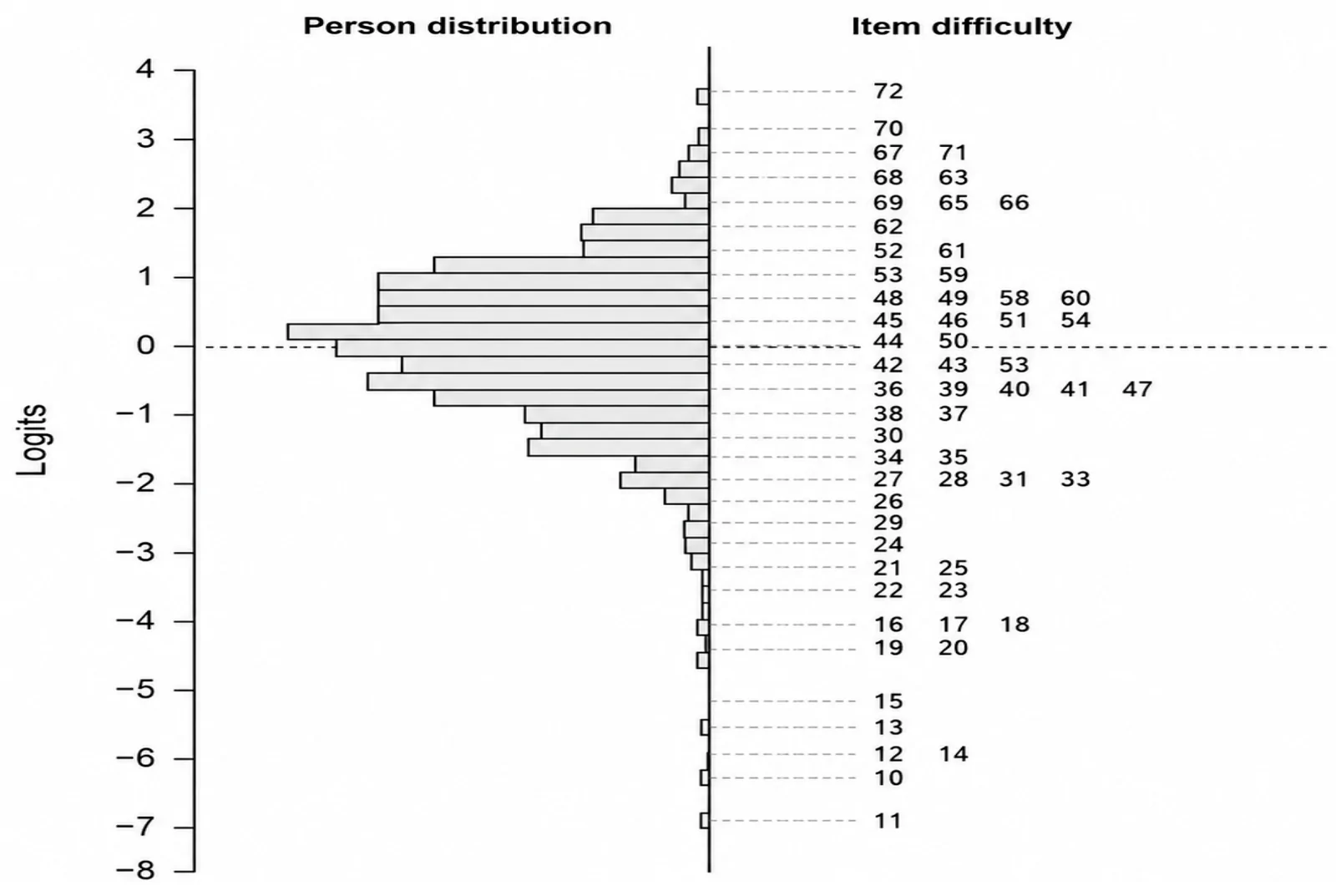
Person–Item Wright Map for the Sensitivity Rasch Model. Note. Person ability estimates (**left**) and item difficulty estimates (**right**) are displayed on the same logit scale. The figure is based on the sensitivity Rasch model, in which post-discontinuation nonadministered responses were treated as missing. Items 1–9 were excluded from Rasch calibration because of pronounced ceiling effects and insufficient response variability.

Person–item targeting was further examined using a Wright map based on the sensitivity Rasch model (Figure 1). The mean person ability estimate was −0.08 logits (SD = 1.23), whereas the mean item difficulty was −0.68 logits, corresponding to a person–item mean difference of 0.60 logits and indicating that the calibrated items were somewhat easier, on average, than the ability level of the sample. Nevertheless, the item difficulties covered a broad range (−7.03 to 3.28 logits). The 95th, 97.5th, and 99th percentiles of the person ability distribution were 1.77, 2.09, and 2.60 logits, respectively, whereas the two most difficult items were located at 3.28 and 2.70 logits. Only two participants (0.18%) had ability estimates exceeding the difficulty of the most difficult item. Overall, the calibrated item set covered most of the observed ability distribution, including the upper end, although item density was lower at the most extreme levels of ability.

Local independence was further examined using Yen’s Q3 and adjusted Q3 (aQ3) residual correlations. In the primary Rasch analysis, the mean absolute aQ3 was 0.042 and the maximum aQ3 was 0.424. Of the 1953 item pairs, 29 showed aQ3 values above 0.20 and eight showed values above 0.30. Several of the larger residual associations involved later items, for which post-discontinuation responses had been coded as incorrect in the primary dataset. In the sensitivity analysis, in which nonadministered responses were treated as missing, the mean absolute aQ3 was 0.054 and the maximum was 0.436. Because the number of jointly administered responses decreased substantially for later item pairs, residual associations were additionally examined descriptively among pairs with at least 200 jointly administered responses. Within this subset, 10 of 1485 pairs had aQ3 values above 0.20 and two had values above 0.30. The largest residual associations were observed between Items 12 and 13 (aQ3 = 0.436) and Items 10 and 13 (aQ3 = 0.345), with both estimates based on 1130 jointly administered responses. Taken together, the residual analyses indicated generally limited local dependence, although elevated residual associations remained for a small number of item pairs.

Table 6 presents the results of the differential item functioning (DIF) analyses across gender and age groups. For gender, nine items (Items 13, 20, 22, 26, 27, 28, 31, 39, and 53) demonstrated nominally significant DIF prior to correction for multiple testing (*p* < .05). Following the Benjamini–Hochberg false discovery rate correction, only Item 26 remained statistically significant (adjusted *p* < .001), whereas the remaining items were no longer significant. For age, three items (Items 17, 35, and 42) demonstrated nominally significant DIF; however, none remained statistically significant after Benjamini–Hochberg correction. The sensitivity analysis, in which post-discontinuation nonadministered responses were treated as missing, yielded a similar pattern. For gender, nine items showed nominally significant DIF, with only Item 26 remaining significant after Benjamini–Hochberg correction, χ^2^(1) = 15.19, adjusted *p* = .006. The absolute difficulty contrast for Item 26 was 0.66 logits, with the item being relatively more difficult for females. The Item 26 finding was also retained in the purified-anchor sensitivity analysis, in which the 54 items without nominal gender DIF in the initial analysis were used as anchors, χ^2^(1) = 14.55, *p* < .001. The corresponding absolute difficulty contrast was 0.65 logits. For age, two items showed nominally significant DIF, but neither remained significant after correction. Overall, there was limited evidence of uniform DIF across gender and age groups under the specified Rasch model, with Item 26 representing the only consistent exception for gender.

Table 7 presents the validity and reliability findings for the NNAT-I. Test–retest reliability was high (Pearson’s *r* = 0.899; ICC[A,1] = 0.894, 95% CI [0.846, 0.928]; SEM = 3.49), as was alternate-form reliability (Pearson’s *r* = 0.906; ICC[A,1] = 0.901, 95% CI [0.854, 0.933]; SEM = 3.15). Convergent validity evidence was supported by strong positive correlations between NNAT-I scores and the Test of Nonverbal Intelligence–Third Edition (TONI-3; *r* = 0.865, 95% CI [0.822, 0.898]; age-adjusted partial *r* = 0.862; *p* < .001) and Raven’s Standard Progressive Matrices (RSPM; *r* = 0.798, 95% CI [0.713, 0.860]; age-adjusted partial *r* = 0.791; *p* < .001). The demographic comparability of the subsamples with the full sample was also examined. Subsample membership was not significantly associated with gender for the test–retest (χ^2^(1) = 3.76, *p* = .052), alternate-form (χ^2^(1) = 0.26, *p* = .608), TONI-3 (χ^2^(1) = 0.39, *p* = .533), or RSPM (χ^2^(1) = 1.09, *p* = .297) subsamples. Age-group distributions did not differ significantly for the test–retest subsample (χ^2^(4) = 7.21, *p* = .125), whereas significant differences were observed for the alternate-form (χ^2^(4) = 10.79, *p* = .029), TONI-3 (χ^2^(4) = 43.40, *p* < .001), and RSPM (χ^2^(4) = 10.97, *p* = .027) subsamples. Grade-level distributions differed significantly for all four subsamples (all *p* ≤ .001). Thus, although the subsamples were broadly comparable to the full sample with respect to gender, differences in age and particularly grade-level composition should be considered when interpreting the reliability and convergent validity findings.

As shown in Table 8, the null random-intercept model indicated that approximately 1.5% of the variance in NNAT-I total scores was attributable to differences between schools (ICC = 0.015). Although between-school differences accounted for only a small proportion of the total variance, the hierarchical structure of the sample justified the use of linear mixed-effects models in subsequent analyses. The final linear mixed-effects model showed that grade level was significantly associated with NNAT-I total scores, F(3, 1037.97) = 13.06, *p* < .001. Parameter estimates indicated that students in Grades 5, 6, and 7 scored significantly lower than those in Grade 8 (all *p* < .001). After controlling for age and school-level clustering, female students scored significantly higher than male students (B = 2.11, *SE* = 0.55, *t* = 3.83, *p* < .001). In contrast, age was not significantly associated with NNAT-I total scores (B = −0.95, *SE* = 0.60, *t* = −1.58, *p* = .114). Residual diagnostics indicated no substantial departures from approximate normality or homoscedasticity. Standardized residuals ranged from −3.08 to 3.10, with only two observations (0.18%) exceeding an absolute value of 3. Given the strong association between chronological age and grade level (Pearson *r* = 0.914; Spearman ρ = 0.933), additional sensitivity models were estimated to examine the stability of their associations with NNAT-I scores. Collinearity diagnostics indicated substantial overlap between age and grade, with an age VIF of 6.08 and tolerance of 0.164. In the age-only mixed-effects model, age was positively associated with NNAT-I scores (*B* = 2.27, *SE* = 0.24, *p* < .001). In the grade-only model, higher grade levels were significantly associated with higher NNAT-I scores (all *p*s < .001). When age and grade were entered simultaneously, the age coefficient changed direction and was no longer statistically significant (*B* = −1.10, *SE* = 0.60, *p* = .067), whereas grade remained significantly associated with NNAT-I scores (overall *p* < .001). This change in the age coefficient indicates that the mutually adjusted age and grade estimates should be interpreted cautiously given their substantial overlap.

## 4. Discussion

Findings from the 1130 participants provide evidence of the psychometric performance of the NNAT-I in this Turkish middle-school sample. The high internal-consistency coefficients indicate strong consistency among the NNAT-I item responses. These results are also generally consistent with findings from the previous NNAT norm study conducted in Türkiye with children aged 5–9 years, which reported high levels of internal consistency, alternate-form reliability, and test–retest reliability (Kavruk & Bildiren, 2022). Importantly, the present study extends this evidence to middle school students aged 10–14 years, an age group not represented in previous Turkish psychometric research on the NNAT-I.

Correlations between NNAT-I scores and scores on the TONI-3 and RSPM provided evidence of convergence with other measures of nonverbal reasoning. In the earlier Turkish study conducted with younger children, strong associations were reported between the NNAT and the CPM, whereas the relationship with the TONI-3 was found to be comparatively lower (Kavruk & Bildiren, 2022). In the present study, correlations with both the RSPM and TONI-3 provide further convergent validity evidence for the NNAT-I in this age group. These associations remained essentially unchanged after adjustment for chronological age, suggesting that the observed convergence was not primarily attributable to age differences within the sample.

Balboni et al. (2010) reported moderate concurrent and predictive validity of the NNAT with Raven measures and found that NNAT scores contributed to the prediction of mathematics achievement. Similarly, the meta-analysis by Lee et al. (2021) showed that the NNAT generally demonstrated moderate associations with a range of external validity criteria. The comparatively strong associations observed in the present study therefore extend convergent validity evidence to Turkish middle school students, although direct comparisons of coefficient magnitude should be made cautiously because of differences in samples, educational contexts, and study designs.

The CFA and item-level analyses provided complementary evidence regarding the internal structure of the NNAT-I. The primary one-factor CFA of Items 10–72 yielded mixed evidence of model fit, with a low scaled RMSEA but inadequate scaled CFI, scaled TLI, and SRMR values. However, model fit improved substantially in the sensitivity analysis restricted to Items 21–65, for which both response categories were adequately represented. This pattern suggests that extreme item response distributions, particularly pronounced ceiling and floor effects, may influence the apparent factorial structure of the instrument. The sensitivity analysis provided more favorable, although not unequivocal, evidence for a predominantly unidimensional structure among items with adequate response variability. The additional structural-nonadministration CFA yielded mixed fit when post-discontinuation items were treated as missing, further indicating that conclusions regarding factorial structure are sensitive to both response distributions and the treatment of structurally nonadministered responses. Item-level analyses similarly indicated that the full test included items spanning a broad range of difficulty. Because Items 1–9 were excluded from the Rasch calibration owing to pronounced ceiling effects and limited response variability, the Rasch-derived difficulty hierarchy applies to Items 10–72 rather than to the complete NNAT-I item set. Most calibrated items demonstrated acceptable Infit statistics, supporting the general ordering of item difficulty, although elevated Outfit values for several items indicated some departures from model expectations. The sensitivity analysis further showed that the relative item-difficulty hierarchy and Infit results were highly stable when post-discontinuation nonadministered responses were treated as missing, whereas absolute difficulty estimates for later items and Outfit statistics were more sensitive to their treatment. This distinction is important because the raw-score scoring convention and item-response calibration serve different purposes. Although the zero-coded model was retained as the primary Rasch analysis because it reflected the NNAT-I raw-score scoring convention and the scoring structure used in the primary analyses, some of these zeros represented structurally nonadministered rather than observed incorrect responses. The reconstructed-response model therefore provides an important complementary psychometric calibration. The high correspondence in relative item difficulty across the two models indicates that the difficulty hierarchy was robust, whereas differences in absolute difficulty estimates and outfit statistics indicate that some Rasch parameters were sensitive to the treatment of nonadministered responses. Accordingly, the two Rasch analyses should be interpreted jointly rather than either coding approach being regarded as definitive for item-response calibration. Taken together, the CFA and Rasch analyses provide evidence regarding the instrument’s internal structure, while also indicating that conclusions concerning strict unidimensionality and item functioning should remain qualified. The residual-dependence analysis provided further information regarding the local independence assumption of the Rasch model. Residual associations were generally limited, although a small number of item pairs showed elevated aQ3 values, most notably Items 12–13 and 10–13. In the primary analysis, several additional elevated associations occurred among later items; these were substantially reduced when post-discontinuation nonadministered responses were treated as missing. This pattern suggests that some of the residual association among later items may reflect the scoring treatment of structurally nonadministered responses rather than substantive item dependence. Nevertheless, the remaining localized residual associations indicate that local independence was not absolute and should be considered when interpreting the Rasch results.

Although nine items demonstrated nominally significant gender-related DIF, only Item 26 remained statistically significant after controlling for multiple comparisons using the Benjamini–Hochberg procedure. This finding was replicated in the sensitivity analysis in which post-discontinuation nonadministered responses were treated as missing; Item 26 again remained significant after multiple-testing correction, with a somewhat smaller difficulty contrast (0.66 logits vs. 0.82 logits in the primary analysis). Similarly, three items showed nominal evidence of age-related DIF, but none remained significant following adjustment for multiple testing; the sensitivity analysis yielded the same substantive conclusion for age, with no items remaining significant after correction. The analyses provided limited evidence of differential item functioning across gender and age groups. The observed group-level differences in NNAT-I scores therefore do not appear to be attributable to widespread gender- or age-related DIF, although the consistent gender-related DIF observed for Item 26 warrants further examination and replication in independent samples.

Recent psychometric research has emphasized the need to evaluate nonverbal measures using multiple sources of evidence rather than relying primarily on internal consistency. For example, Benson et al. (2020) used latent-variable methods to examine the dimensional structure and measurement invariance of the UNIT2, illustrating that high reliability does not necessarily justify separate interpretation of theoretically proposed dimensions or guarantee equivalent measurement across demographic groups. Turkish studies have likewise combined reliability and validity evidence when evaluating nonverbal measures (Bildiren et al., 2021; Bildiren et al., 2025; Kavruk & Bildiren, 2022; Ozdemir et al., 2026). The present study extends this approach by combining classical reliability and convergent validity evidence with CFA, Rasch modeling, local-dependence analysis, and DIF assessment in Turkish middle school students.

The differential associations of chronological age and grade level with NNAT-I performance warrant careful interpretation. Chronological age and grade level were strongly correlated (*r* = 0.914), reflecting their substantial overlap in this school-based sample. The sensitivity analyses further showed that chronological age was positively associated with NNAT-I performance when examined without grade level, whereas its coefficient changed direction and was no longer statistically significant when age and grade were entered simultaneously. In contrast, grade level remained significantly associated with NNAT-I performance in the mutually adjusted model. This pattern indicates that the coefficients for age and grade should not be interpreted as clearly separable developmental or educational effects. Given the strong overlap between these predictors and the cross-sectional design, the observed grade-level differences may reflect a combination of developmental, educational, cohort, selection, or other factors associated with school progression. Accordingly, the findings should be interpreted as cross-sectional associations and do not establish that formal schooling itself improves nonverbal reasoning.

The present findings should be considered in the context of the ongoing debate regarding the extent to which nonverbal ability tests reduce cultural and linguistic influences on cognitive assessment. The NNAT was developed to minimize language demands by relying on visual matrix reasoning rather than reading, writing, or verbal responses (Lee et al., 2021). However, this design should not be interpreted as rendering the test completely culture-free. Meta-analytic evidence (Lee et al., 2021) and empirical findings (Carman et al., 2020) suggest that nonverbal measures alone do not eliminate disparities in gifted identification and that assessment outcomes may also be influenced by factors such as local norms, identification criteria, and educational context. These results are consistent with using the NNAT-I as one component of a comprehensive assessment process rather than as a standalone basis for educational decision making. They also reinforce the importance of establishing local psychometric evidence before widely used nonverbal ability measures are implemented in new educational contexts.

Mean NNAT-I scores were descriptively similar across districts representing different socioeconomic development levels (SEGE). In an additional sensitivity analysis, SEGE was included as a school-level fixed predictor in the mixed-effects model and was not significantly associated with NNAT-I performance, *F*(3, 2.0) = 0.23, *p* = .872. However, this finding should be interpreted cautiously because the study included only six school clusters, and three of the four SEGE strata were represented by a single school, providing limited independent information for estimating contextual socioeconomic differences. Accordingly, the nonsignificant SEGE effect should not be interpreted as evidence that socioeconomic context is unrelated to nonverbal reasoning. Although previous research suggests that nonverbal intelligence may be less strongly associated with socioeconomic inequalities than verbally mediated abilities (Madhushanthi et al., 2020), the present data do not provide a sufficiently precise test of this proposition. Further research incorporating individual-level socioeconomic indicators and a larger number of schools within each socioeconomic stratum is needed to clarify the relationship between socioeconomic context and NNAT-I performance.

Considering that nonverbal tests cannot be regarded as entirely independent of cultural context, the present study also highlights the importance of future normative research on the NNAT-I in Türkiye. Vista and Grantham (2009) demonstrated that although Filipino students obtained NNAT mean scores comparable to those of the U.S. normative sample, score distributions differed substantially, particularly at the lower and upper ends of the distribution. This finding suggests that similarity in mean scores alone is insufficient for cross-cultural norm transfer and that locally established normative data may be particularly important in decision-making processes based on upper percentile ranges, such as gifted identification. Similarly, Vista (2006) emphasized that relying solely on mean score comparisons may be misleading and that differences in stanine distributions may have important implications for the classification of high-performing students. From this perspective, the present study provides regional psychometric evidence for Turkish middle school students that may inform future normative research; however, the present sample should not be interpreted as establishing national Turkish norms for the NNAT-I.

The importance of local norms, particularly in decisions based on upper percentile ranges, also necessitates careful consideration of the present findings in relation to the identification of gifted students. Naglieri and Ford (2003) argued that the NNAT produced comparable rates of high scores across different ethnic groups and therefore might increase minority students’ access to gifted education programs. However, Lohman (2005) noted that such interpretations should be evaluated carefully in terms of sample representativeness and socioeconomic distribution. Similarly, the meta-analysis conducted by Lee et al. (2021) suggested that although the NNAT contributes to the identification of underrepresented groups to some extent, these groups remain insufficiently represented in gifted education programs. Accordingly, while the present results provide evidence for the potential utility of the NNAT-I in screening and assessment processes in Türkiye, the test should preferably be used as part of a multi-criteria evaluation system rather than as a standalone instrument in high-stakes decisions such as gifted identification.

The principal scientific contribution of the present study lies not simply in replicating previous evidence of NNAT-I reliability and validity, but in extending its psychometric evaluation to Turkish students aged 10–14 years and integrating classical reliability and convergent validity evidence with CFA, Rasch modeling, DIF analysis, and multilevel modeling. This combined approach provides age-specific, item-level, factorial, and group-related evidence that was not available in the previous Turkish study of younger children. At the same time, the mixed primary CFA fit, extreme response distributions for some items, and the limited DIF findings demonstrate the value of evaluating the instrument beyond conventional reliability coefficients alone.

The present study extends the available psychometric evidence for the NNAT-I in Turkish middle school students aged 10–14 years. The study provides qualified evidence regarding the reliability, convergent validity, and psychometric functioning of the NNAT-I in this age group. These results are consistent with its use as one component of a comprehensive assessment process rather than as a standalone basis for educational decision making.

### 4.1. Limitations

The findings of this study should be interpreted in light of several limitations. First, the sample was drawn from schools in a single province (Aydın), which may limit the generalizability of the results to other geographical and sociocultural regions of Türkiye. Second, although the hierarchical structure of the data was addressed using linear mixed-effects models, the between-school variance was small (ICC = 0.015). Accordingly, the findings should primarily be interpreted at the individual level. In addition, SEGE reflected district-level socioeconomic development rather than individual socioeconomic status. Although SEGE was examined as a school-level fixed predictor in an additional sensitivity analysis, the study included only six school clusters, and three of the four SEGE strata were represented by a single school. This limited the precision with which independent SEGE effects could be estimated and made it difficult to distinguish contextual differences associated with SEGE from characteristics specific to individual schools. Accordingly, the absence of a statistically significant SEGE effect should be interpreted cautiously. Future studies should include a larger number of schools within each socioeconomic stratum and individual-level socioeconomic indicators to permit a more robust evaluation of socioeconomic differences in NNAT-I performance. The reliability and convergent-validity subsamples were broadly comparable to the full sample in terms of gender but differed in age and, particularly, grade-level composition. Accordingly, findings from these subsample-based analyses should be interpreted with caution regarding their generalizability to the full sample. The Turkish norming and standardization evidence cited for the TONI-3 was based on children aged 6–11 years and therefore does not cover the full age range of the present sample (10–14 years). Although the observed NNAT-I–TONI-3 association remained strong after adjustment for age, the available local validation evidence does not extend to the oldest participants in the present sample. Accordingly, the convergent-validity findings involving the TONI-3 should be interpreted with this limitation in mind. The alternate-form reliability assessment used a fixed administration order (Form A followed by Form B) without counterbalancing; therefore, potential form effects cannot be fully distinguished from order or practice effects. Third, although the CFA, Rasch, and DIF analyses provided complementary evidence regarding the internal structure and item functioning of the NNAT-I, the findings did not provide unequivocal support for strict unidimensionality. The primary one-factor CFA yielded mixed fit, whereas fit improved substantially when items with extreme response distributions were excluded in the response-distribution sensitivity analysis; however, the additional structural-nonadministration CFA also yielded mixed fit when post-discontinuation nonadministered items were treated as missing. In addition, residual-dependence analyses identified elevated associations for a small number of item pairs, indicating that local independence was not absolute. These findings warrant some caution when interpreting the unidimensional Rasch model and suggest that the dimensional structure of the NNAT-I should be examined further using alternative latent-variable and more flexible IRT models. Formal measurement invariance across groups was not evaluated using multiple-group CFA or other comprehensive invariance-testing approaches. Although only Item 26 demonstrated statistically significant gender-related DIF after adjustment for multiple comparisons, and this finding was retained in the sensitivity analysis, it should be replicated in independent samples before firm conclusions are drawn regarding its stability. Finally, although the sensitivity analyses indicated that the relative item-difficulty hierarchy and Infit results were generally robust to the treatment of post-discontinuation responses, absolute difficulty estimates for later items and Outfit statistics were more sensitive to this scoring decision.

### 4.2. Implications

Further research should examine the psychometric properties and normative structure of the NNAT-I in larger and more diverse samples drawn from different sociocultural regions of Türkiye. Additional studies should also investigate differential item functioning across other demographic characteristics, including socioeconomic strata, geographic regions, and special populations. Formal evaluations of measurement invariance using multiple-group CFA or complementary IRT-based approaches would further strengthen the evidence regarding score comparability across groups. From a practical perspective, the NNAT-I may be useful as a screening instrument in assessment contexts where reducing language demands is relevant. However, for high-stakes decisions such as gifted identification, it is more appropriately used as one component of a comprehensive, multi-criteria assessment process rather than as a standalone measure.

## 5. Conclusions

The present study provides psychometric evidence regarding the reliability, convergent validity, and item functioning of the NNAT-I among Turkish middle school students. Evidence from classical test theory, confirmatory factor analysis, Rasch modeling, and differential item functioning analyses generally indicates adequate psychometric performance in this population, while also identifying qualifications regarding strict unidimensionality, localized residual dependence, and the treatment of post-discontinuation responses. Further research is warranted to evaluate measurement invariance formally and to establish normative data across broader and more diverse samples. The present findings nevertheless indicate that the NNAT-I may serve as a useful component of comprehensive cognitive assessment. Consistent with current recommendations in the gifted education literature, NNAT-I scores should be interpreted alongside multiple sources of evidence rather than used as a standalone basis for high-stakes educational decisions.

## Figures and Tables

**Table 1:** Demographic Characteristics of the Participants.

Variables	*n* (%)
Gender	
Female	560 (49.6%)
Male	570 (50.4%)
Age, years, M ± SD	11.74 ± 1.16
Age (Years, Months)	
10 y 0 m–10 y 11 m	203 (18.0%)
11 y 0 m–11 y 11 m	334 (29.6%)
12 y 0 m–12 y 11 m	274 (24.2%)
13 y 0 m–13 y 11 m	256 (22.7%)
14 y 0 m–14 y 6 m	63 (5.6%)
Grade	
Grade 5	333 (29.5%)
Grade 6	285 (25.2%)
Grade 7	254 (22.5%)
Grade 8	258 (22.8%)
Development Level (SEGE)	
1st Level	503 (44.5%)
2nd Level	231 (20.4%)
3rd Level	256 (22.7%)
4th Level	140 (12.4%)

**Table 2:** Descriptive statistics for the variables.

Variables	*n*	*M ± SD*	Min	Max
Gender				
Female	560	41.42 ± 9.64	15.00	68.00
Male	570	39.10 ± 9.91	12.00	70.00
Age (years, months)				
10 y 0 m–10 y 11 m	203	36.71 ± 8.78	14.00	64.00
11 y 0 m–11 y 11 m	334	38.63 ± 9.59	12.00	65.00
12 y 0 m–12 y 11 m	274	41.05 ± 9.96	15.00	70.00
13 y 0 m–13 y 11 m	256	43.28 ± 9.58	19.00	68.00
14 y 0 m–14 y 6 m	63	44.57 ± 9.40	29.00	63.00
Grade				
5th grade	333	35.99 ± 8.78	14.00	64.00
6th grade	285	40.15 ± 9.61	12.00	65.00
7th grade	254	41.78 ± 9.68	15.00	70.00
8th grade	258	44.38 ± 9.39	23.00	68.00
Development Level (SEGE)				
1st Level	503	40.20 ± 9.96	14.00	70.00
2nd Level	231	40.28 ± 9.30	17.00	62.00
3rd Level	256	40.70 ± 10.37	15.00	67.00
4th Level	140	39.61 ± 9.28	12.00	64.00

**Table 3:** McDonald’s Omega and Cronbach’s Alpha Coefficients by Gender, Age, Grade Level, and Socioeconomic Development Level.

Variables	McDonald Omega	Cronbach’s Alpha
Gender		
Female	—	0.93
Male	—	0.93
Age (Years, Months)		
10 y 0 m–10 y 11 m	—	0.92
11 y 0 m–11 y 11 m	0.94	0.93
12 y 0 m–12 y 11 m	0.94	0.93
13 y 0 m–13 y 11 m	0.93	0.93
14 y 0 m–14 y 6 m	0.93	0.92
Total	0.94	0.93
Grade		
5th grade	0.92	0.92
6th grade	0.93	0.93
7th grade	0.94	0.93
8th grade	0.93	0.92
Total	0.94	0.93
Development Level (SEGE)		
1st Level	0.94	0.94
2nd Level	0.93	0.92
3rd Level	0.94	0.94
4th Level	0.93	0.93
Total	0.94	0.93

Note. McDonald’s ω could not be estimated for the female, male, and 10-year-old groups due to computational convergence problems associated with perfectly correlated items. Because the NNAT-I items were dichotomously scored (0 = incorrect, 1 = correct), Cronbach’s alpha coefficients were interpreted as KR-20 reliability estimates.

**Table 4:** Confirmatory Factor Analysis Fit Indices for the NNAT-I.

Model	Items *(n)*	CFI	TLI	RMSEA	SRMR	Scaled CFI	Scaled TLI	Scaled RMSEA
Primary one-factor model	10–72 (63)	0.924	0.921	0.069	0.169	0.850	0.845	0.039
Sensitivity one-factor model	21–65 (45)	0.981	0.980	0.044	0.097	0.951	0.948	0.041
Structural-nonadministration sensitivity model	21–65 (45)	0.924	0.921	0.020	0.108	0.897	0.892	0.017

Note. The response-distribution sensitivity analysis included Items 21–65, for which both response categories represented at least 5% of observations. In the structural-nonadministration sensitivity analysis, the four observed incorrect responses triggering discontinuation were retained as incorrect, whereas subsequent nonadministered items were treated as missing. The corresponding model including Items 10–72 did not converge because of sparse pairwise coverage and empty bivariate response cells; therefore, the structural-nonadministration sensitivity CFA was estimated using the Items 21–65 subset. CFI = comparative fit index; TLI = Tucker–Lewis index; RMSEA = root mean square error of approximation; SRMR = standardized root mean square residual.

**Table 5:** Summary of Classical Item Statistics and Rasch Model Results for the NNAT-I.

Analysis	Statistic/Range	Interpretation
Item Difficulty (*p*)	0.01–1.00	Items ranged from very easy to very difficult
Corrected Item–Total Correlation	−0.005–0.643	Item discrimination varied across items; 11 items had coefficients below 0.20.
**Primary Rasch Analysis**		
Rasch Item Difficulty (logit)	−7.89 to 6.04	Broad hierarchical difficulty structure across the test
Infit range	0.76–1.23	All 63 items were within the prespecified 0.70–1.30 range
Outfit range	0.20–6.51	17 of 63 items were within the prespecified 0.70–1.30 range
EAP Reliability	0.941	Excellent measurement precision
**Sensitivity Rasch Analysis**		
Rasch Item Difficulty (logit)	−7.03 to 3.28	The difficulty range narrowed when nonadministered responses were treated as missing
Item-difficulty correspondence	*r* = 0.991	Relative item-difficulty ordering was highly stable
Mean absolute difficulty difference	0.90 logits	Absolute estimates changed, particularly for later items
Infit within 0.70–1.30	63/63	Infit conclusions were unchanged
Outfit within 0.70–1.30	56/63	Outfit fit improved substantially when nonadministered responses were treated as missing
EAP Reliability	0.855	Measurement reliability remained high

Note. Rasch item-difficulty estimates are reported in logits. Items 1–9 were excluded from Rasch calibration because of pronounced ceiling effects and insufficient response variability; therefore, Rasch analyses included Items 10–72. In the sensitivity analysis, nonadministered items following the discontinue point were treated as missing. Detailed primary and sensitivity Rasch item statistics are provided in Supplementary Table S2.

**Table 6:** Differential Item Functioning Analyses across Gender and Age Groups in the Primary Analysis.

Comparison	Item	χ^2^	df	*p*	BH-Adjusted *p*	Difficulty Contrast (Logits)
Gender	Item 13	4.62	1	.032	.251	1.86 (M)
	Item 20	5.36	1	.021	.251	0.88 (M)
	Item 22	4.07	1	.044	.306	0.50 (F)
	**Item 26**	**21.50**	**1**	**<.001**	**<.001**	**0.82 (F)**
	Item 27	8.02	1	.005	.112	0.49 (F)
	Item 28	4.60	1	.032	.251	0.37 (F)
	Item 31	4.79	1	.029	.251	0.37 (M)
	Item 39	5.11	1	.024	.251	0.34 (M)
	Item 53	7.77	1	.005	.112	0.51 (M)
Age	Item 17	10.41	4	.034	.563	2.40 (age 14)
	Item 35	14.08	4	.007	.444	0.92 (age 13)
	Item 42	10.91	4	.028	.563	1.06 (age 14)

Note. χ^2^ = likelihood-ratio chi-square statistic obtained from the multiple-group Rasch model; BH = Benjamini–Hochberg adjustment. For gender, difficulty contrast represents the absolute difference between female and male item difficulty estimates; F and M indicate the group for which the item was relatively more difficult. For age, difficulty contrast represents the range between the highest and lowest age-group-specific item difficulty estimates, and the age in parentheses indicates the group for which the item was most difficult. Only Item 26 remained statistically significant after Benjamini–Hochberg correction. Sensitivity DIF analyses, in which post-discontinuation nonadministered responses were treated as missing, yielded the same substantive conclusions and are reported in the text.

**Table 7:** Validity and Reliability Results for the NNAT-I.

Analysis Type	Compared Measures	*n*	*M*	*SD*	*r*	Age-Adjusted Partial Correlation (*r*)	ICC (A,1)	95% CI	SEM
Test–Retest Reliability	Initial Application	105	42.25	10.72	0.899 *	–	0.894	[0.846, 0.928]	3.49
	After 4 Weeks		43.37	11.27					
Alternate-Form Reliability	Form A	107	38.80	10.01	0.906 *	–	0.901	[0.854, 0.933]	3.15
	Form B		37.69	9.62					
Convergent Validity	NNAT-I	171	41.83	10.13	0.865 *	0.862	–	[0.822, 0.898]	--
	TONI-3		29.75	7.64					
	NNAT-I	98	40.68	9.80	0.798 *	0.791	–	[0.713, 0.860]	--
	RSPM		42.05	8.73					

Note. ICC = intraclass correlation coefficient, estimated using a two-way mixed-effects, absolute-agreement, single-measure model [ICC(A,1)]. CI = confidence interval. For test–retest and alternate-form reliability, 95% CIs refer to the ICC estimates; for convergent validity analyses, 95% CIs refer to the Pearson correlation coefficients. SEM = standard error of measurement, calculated as *SD* × √(1 − *ICC*), where *SD* represents the standard deviation of the initial administration (test–retest) or Form A (alternate-form reliability). Age-adjusted partial correlations were calculated by controlling for chronological age. * *p* < .001 (two-tailed).

**Table 8:** Null random-intercept model and linear mixed-effects model predicting NNAT-I total scores.

Predictor	Estimate (B)	*SE*	*t*/*F*	*p*	95% CI
**Null model**					
School-level variance (τ^2^)	1.475	0.586	—	—	—
Residual variance (σ^2^)	95.805	4.072	—	—	—
Intraclass correlation coefficient (ICC)	**0.015**	—	—	—	—
**Final mixed-effects model**					
**Fixed effects**					
Intercept	55.88	8.01	6.97	<.001	[40.18, 71.58]
Age	−0.95	0.60	−1.58	.114	[−2.13, 0.23]
Grade level †	—	—	*F*(3, 1037.97) = 13.06	<.001	—
Grade 5	−11.00	1.85	−5.94	<.001	[−14.63, −7.37]
Grade 6	−5.96	1.40	−4.26	<.001	[−8.70, −3.22]
Grade 7	−3.56	1.00	−3.55	<.001	[−5.52, −1.60]
Gender (Female)	2.11	0.55	3.83	<.001	[1.03, 3.19]
**Random effects**					
School-level variance (τ^2^)	0.681	0.868	—	—	—
Residual variance (σ^2^)	85.391	3.613	—	—	—
**Model fit**			**Value**		
−2 Restricted Log Likelihood			8226.56		
Akaike Information Criterion (AIC)			8230.56		
Bayesian Information Criterion (BIC)			8240.60		

Note. The null random-intercept model was estimated to quantify the proportion of variance attributable to differences between schools, and the intraclass correlation coefficient (ICC) was calculated from this model. The final linear mixed-effects model included school as a random intercept. Grade 8 served as the reference category for grade level, and male students served as the reference category for gender. † Overall Type III test for the effect of grade level.

## Data Availability

The datasets generated and analyzed during the current study are not publicly available due to ethical and institutional restrictions but are available from the corresponding author upon reasonable request.

## Supplementary Materials

The following supporting information can be downloaded at: https://www.mdpi.com/article/10.3390/jintelligence14090209/s1, Table S1. Standardized Factor Loadings and Threshold Parameters for the Primary One-Factor CFA of the NNAT-I. Table S2. Primary and Sensitivity Rasch Item Difficulty and Fit Statistics for the NNAT-I.

## Abbreviations

AIC	Akaike Information Criterion
BIC	Bayesian Information Criterion
CFA	Confirmatory Factor Analysis
CI	Confidence Interval
CTT	Classical Test Theory
DIF	Differential Item Functioning
EAP	Expected A Posteriori
FDR	False Discovery Rate
ICC	Intraclass Correlation Coefficient
IRT	Item Response Theory
KR-20	Kuder–Richardson Formula 20
MoIT	Ministry of Industry and Technology
MoNE	Ministry of National Education
NNAT-I	Naglieri Nonverbal Ability Test–Individual Form
RSPM	Raven’s Standard Progressive Matrices
SE	Standard Error
SEGE	Socio-Economic Development Ranking Research
SEM	Standard Error of Measurement
SPSS	Statistical Package for the Social Sciences
TAM	Test Analysis Modules
TLI	Tucker–Lewis Index
TONI-3	Test of Nonverbal Intelligence–Third Edition
