# Supplementary material for: Psychometric Evaluation of the Naglieri Nonverbal Ability Test (NNAT-I) in Turkish Middle School Students Aged 10–14 Years
Source: J Intell. 2026 Sep 1;14(9):209. doi: 10.3390/jintelligence14090209 (PMC13608390; doi:10.3390/jintelligence14090209)
Supplement: Supplementary file 1 [file jintelligence-14-00209-s001.zip › jintelligence-4496746-supplementary.pdf]

Supplementary Table S1. Standardized Factor Loadings and Threshold Parameters for the Primary One-Factor CFA of the NNAT-I.

Item	Standardized_Loading	SE	z	p
Item 10	0.232	0.068	3.417	< .001
Item 11	0.070	0.028	2.532	.011
Item 12	0.229	0.069	3.322	< .001
Item 13	0.373	0.090	4.134	< .001
Item 14	0.174	0.072	2.435	.015
Item 15	0.413	0.090	4.599	< .001
Item 16	0.496	0.071	6.975	< .001
Item 17	0.529	0.063	8.405	< .001
Item 18	0.575	0.070	8.251	< .001
Item 19	0.447	0.085	5.283	< .001
Item 20	0.679	0.071	9.581	< .001
Item 21	0.534	0.048	11.024	< .001
Item 22	0.556	0.050	11.049	< .001
Item 23	0.571	0.059	9.683	< .001
Item 24	0.425	0.046	9.283	< .001
Item 25	0.466	0.051	9.188	< .001
Item 26	0.524	0.039	13.542	< .001
Item 27	0.539	0.036	14.900	< .001
Item 28	0.490	0.038	12.954	< .001
Item 29	0.555	0.043	13.025	< .001
Item 30	0.483	0.035	13.649	< .001
Item 31	0.529	0.038	14.005	< .001
Item 32	0.550	0.035	15.746	< .001
Item 33	0.650	0.035	18.850	< .001
Item 34	0.594	0.031	19.485	< .001
Item 35	0.569	0.034	16.876	< .001
Item 36	0.708	0.025	28.290	< .001
Item 37	0.711	0.027	26.732	< .001
Item 38	0.707	0.024	29.054	< .001
Item 39	0.761	0.023	32.848	< .001
Item 40	0.678	0.027	25.456	< .001
Item 41	0.655	0.027	24.323	< .001
Item 42	0.587	0.028	21.041	< .001
Item 43	0.792	0.021	38.346	< .001
Item 44	0.719	0.023	31.144	< .001
Item 45	0.674	0.024	27.517	< .001
Item 46	0.695	0.024	29.458	< .001
Item 47	0.843	0.020	41.920	< .001
Item 48	0.764	0.020	37.540	< .001
Item 49	0.752	0.021	36.589	< .001
Item 50	0.772	0.020	37.982	< .001

Item 51	0.820	0.018	46.807	< .001
Item 52	0.854	0.017	50.186	< .001
Item 53	0.793	0.019	42.564	< .001
Item 54	0.836	0.018	47.071	< .001
Item 55	0.749	0.021	35.557	< .001
Item 56	0.767	0.020	38.147	< .001
Item 57	0.784	0.019	41.299	< .001
Item 58	0.804	0.019	42.704	< .001
Item 59	0.789	0.019	40.802	< .001
Item 60	0.862	0.015	58.058	< .001
Item 61	0.797	0.019	41.443	< .001
Item 62	0.758	0.022	34.099	< .001
Item 63	0.730	0.026	28.012	< .001
Item 64	0.714	0.026	27.238	< .001
Item 65	0.715	0.027	26.314	< .001
Item 66	0.713	0.028	25.898	< .001
Item 67	0.725	0.034	21.453	< .001
Item 68	0.767	0.028	27.373	< .001
Item 69	0.836	0.024	34.697	< .001
Item 70	0.676	0.037	18.247	< .001
Item 71	0.753	0.038	19.780	< .001
Item 72	0.631	0.057	11.098	< .001

Note. Standardized factor loadings and threshold parameters are reported for Items 10–72 included in the primary one-factor confirmatory factor analysis. The model was estimated using the WLSMV estimator for dichotomously scored items. SE = standard error.

Supplementary Table S2. Primary and Sensitivity Rasch Item Difficulty and Fit Statistics for the NNAT-I.

Item	Classical Difficulty (p)	Corrected Item-Total r	Primary Difficulty (logit)	Primary SE	Sensitivity Difficulty (logit)	Sensitivity SE	Primary Infit MnSq	Sensitivity Infit MnSq	Primary Outfit MnSq	Sensitivity Outfit MnSq	Primary Infit t	Sensitivity Infit t	Primary Outfit t	Sensitivity Outfit t	Administered, n
Item 1	1														1130
Item 2	1														1130
Item 3	0.999	0.061													1130
Item 4	0.999	0.013													1130
Item 5	1														1130
Item 6	1														1130
Item 7	0.999	-0.005													1130
Item 8	1														1130
Item 9	1														1130
Item 10	0.995	0.118	-6.737	0.424	-5.91	0.414	1.051	0.983	0.428	0.318	0.257	0.089	-1.867	-2.296	1130
Item 11	0.998	0.085	-7.893	0.718	-7.028	0.711	1.026	0.996	0.129	0.181	0.262	0.227	-1.991	-1.671	1130
Item 12	0.994	0.119	-6.57	0.395	-5.751	0.384	1.061	0.98	1.245	0.449	0.286	0.064	0.741	-1.832	1130
Item 13	0.99	0.15	-6.069	0.32	-5.282	0.308	1.022	0.975	0.826	0.569	0.167	0.004	-0.663	-1.73	1130
Item 14	0.994	0.112	-6.57	0.395	-5.751	0.384	1.001	0.973	2.675	0.707	0.117	0.046	3.285	-0.794	1130
Item 15	0.988	0.176	-5.879	0.296	-5.107	0.285	0.916	0.914	0.909	0.559	-0.268	-0.254	-0.349	-1.97	1130
Item 16	0.958	0.211	-4.32	0.166	-3.702	0.156	1.042	0.937	2.573	1.052	0.366	-0.46	8.764	0.414	1130
Item 17	0.958	0.221	-4.293	0.164	-3.691	0.156	1.112	0.98	1.688	0.797	0.898	-0.117	4.577	-1.684	1129
Item 18	0.963	0.231	-4.465	0.174	-3.846	0.166	1.09	0.969	1.586	0.741	0.689	-0.182	3.676	-2.022	1129
Item 19	0.969	0.186	-4.696	0.189	-4.079	0.183	0.981	0.95	6.512	1.552	-0.077	-0.276	17.364	3.034	1128
Item 20	0.963	0.277	-4.465	0.174	-3.885	0.169	0.902	0.887	2.194	0.688	-0.703	-0.78	6.482	-2.44	1127
Item 21	0.904	0.291	-3.169	0.116	-2.704	0.109	1.096	0.958	1.969	0.949	1.219	-0.517	9.791	-0.649	1127
Item 22	0.916	0.285	-3.367	0.122	-2.88	0.116	1.08	0.958	1.891	0.917	0.951	-0.477	8.345	-0.984	1127
Item 23	0.935	0.279	-3.734	0.137	-3.258	0.134	1.022	0.973	2.577	0.926	0.248	-0.229	11.186	-0.703	1122
Item 24	0.854	0.262	-2.539	0.098	-2.171	0.093	1.211	1.061	2.39	1.193	3.338	1.016	16.941	3.024	1118

Item 25	0.897	0.269	-3.078	0.113	-2.681	0.11	1.094	1.022	2.588	1.221	1.248	0.301	14.931	2.623	1116
Item 26	0.797	0.347	-1.992	0.088	-1.694	0.082	1.171	1.007	1.746	1.025	3.378	0.166	12.668	0.524	1114
Item 27	0.781	0.368	-1.85	0.085	-1.575	0.08	1.145	0.982	1.5	0.947	3.024	-0.396	9.471	-1.215	1112
Item 28	0.779	0.338	-1.835	0.085	-1.59	0.081	1.204	1.043	1.765	1.051	4.212	0.956	13.713	1.125	1102
Item 29	0.827	0.363	-2.258	0.092	-1.987	0.09	1.052	0.988	2.445	1.082	0.98	-0.196	19.461	1.426	1100
Item 30	0.7	0.356	-1.248	0.078	-1.074	0.074	1.23	1.038	1.824	1.08	5.622	1.09	17.431	2.23	1094
Item 31	0.761	0.373	-1.694	0.083	-1.491	0.08	1.122	1.025	1.882	1.091	2.72	0.571	16.175	2.043	1087
Item 32	0.727	0.396	-1.442	0.08	-1.286	0.077	1.13	1.02	1.625	1.05	3.104	0.503	13.077	1.251	1078
Item 33	0.758	0.451	-1.673	0.083	-1.549	0.082	0.995	0.978	1.463	0.949	-0.095	-0.472	9.356	-1.108	1067
Item 34	0.635	0.443	-0.822	0.075	-0.763	0.072	1.127	0.997	1.34	1.006	3.502	-0.091	8.756	0.199	1056
Item 35	0.684	0.42	-1.139	0.077	-1.087	0.076	1.117	1.041	1.57	1.08	3.04	1.109	13.063	2.13	1043
Item 36	0.588	0.54	-0.524	0.074	-0.546	0.071	0.995	0.916	0.949	0.855	-0.145	-3.03	-1.538	-5.349	1026
Item 37	0.588	0.536	-0.529	0.074	-0.579	0.072	0.988	0.95	1.097	0.924	-0.341	-1.719	2.778	-2.652	1009
Item 38	0.514	0.544	-0.08	0.073	-0.192	0.07	1.003	0.927	0.972	0.897	0.101	-2.933	-0.839	-4.181	987
Item 39	0.532	0.586	-0.186	0.073	-0.326	0.072	0.912	0.907	0.877	0.874	-2.694	-3.532	-3.839	-4.869	966
Item 40	0.492	0.528	0.052	0.073	-0.181	0.073	1.021	0.993	1.106	1.005	0.623	-0.266	3.048	0.201	913
Item 41	0.487	0.506	0.084	0.073	-0.21	0.074	1.059	1.046	1.171	1.072	1.727	1.65	4.824	2.567	884
Item 42	0.427	0.453	0.442	0.074	0.08	0.074	1.177	1.113	1.186	1.134	4.849	4.302	5.072	5.069	849
Item 43	0.428	0.612	0.437	0.074	0.024	0.076	0.888	0.949	0.789	0.924	-3.338	-1.98	-6.573	-2.984	826
Item 44	0.346	0.546	0.955	0.076	0.509	0.076	0.996	0.995	0.866	0.991	-0.092	-0.207	-3.767	-0.336	808
Item 45	0.299	0.508	1.271	0.078	0.77	0.078	1.046	1.013	0.903	1.031	1.153	0.472	-2.509	1.132	776
Item 46	0.288	0.517	1.352	0.079	0.766	0.08	1.026	1.037	0.861	1.047	0.643	1.358	-3.604	1.701	731
Item 47	0.404	0.643	0.584	0.074	-0.286	0.088	0.811	0.995	0.78	1.025	-5.729	-0.129	-6.774	0.7	668
Item 48	0.245	0.556	1.665	0.083	0.93	0.085	0.918	0.975	0.717	0.987	-1.916	-0.854	-7.215	-0.442	640
Item 49	0.232	0.544	1.77	0.084	0.968	0.087	0.935	0.993	0.687	0.99	-1.482	-0.23	-7.865	-0.335	614
Item 50	0.265	0.569	1.512	0.081	0.621	0.088	0.912	1.031	0.702	1.036	-2.166	1.114	-7.942	1.305	590
Item 51	0.258	0.605	1.571	0.082	0.639	0.09	0.84	0.969	0.589	0.946	-3.967	-1.107	-11.415	-1.976	567
Item 52	0.285	0.632	1.37	0.079	0.119	0.098	0.805	1.001	0.601	1.008	-5.147	0.041	-11.544	0.227	501
Item 53	0.193	0.558	2.097	0.089	1.007	0.097	0.873	0.98	0.571	0.975	-2.673	-0.65	-10.327	-0.802	480

Item 54	0.222	0.595	1.848	0.085	0.632	0.099	0.823	1.014	0.581	1.037	-4.089	0.466	-10.819	1.216	463
Item 55	0.15	0.493	2.517	0.097	1.419	0.104	0.938	1.033	0.584	1.053	-1.087	0.873	-8.573	1.383	442
Item 56	0.148	0.504	2.536	0.097	1.369	0.105	0.912	1.034	0.547	1.054	-1.561	0.931	-9.439	1.436	425
Item 57	0.153	0.524	2.48	0.096	1.184	0.108	0.889	1.042	0.505	1.049	-2.015	1.231	-10.775	1.414	387
Item 58	0.153	0.52	2.48	0.096	1.088	0.111	0.887	1.065	0.529	1.083	-2.055	1.873	-10.123	2.374	363
Item 59	0.139	0.511	2.632	0.099	1.14	0.117	0.88	1.052	0.485	1.061	-2.072	1.436	-10.706	1.67	329
Item 60	0.144	0.565	2.574	0.098	0.899	0.121	0.768	0.962	0.409	0.942	-4.291	-1.07	-13.169	-1.668	308
Item 61	0.104	0.475	3.058	0.11	1.5	0.127	0.849	1.025	0.45	1.05	-2.219	0.585	-9.807	1.158	290
Item 62	0.084	0.432	3.363	0.12	1.786	0.135	0.908	1.053	0.437	1.066	-1.142	0.991	-8.875	1.226	269
Item 63	0.062	0.384	3.772	0.136	2.194	0.148	0.911	1.034	0.421	1.073	-0.904	0.497	-7.668	1.03	253
Item 64	0.064	0.366	3.735	0.135	2.043	0.15	0.95	1.123	0.489	1.233	-0.496	1.836	-6.595	3.346	231
Item 65	0.052	0.348	3.991	0.147	2.088	0.165	0.945	1.11	0.428	1.173	-0.481	1.483	-6.814	2.275	194
Item 66	0.048	0.337	4.103	0.152	1.998	0.175	0.966	1.125	0.406	1.149	-0.265	1.656	-6.792	1.957	166
Item 67	0.029	0.299	4.7	0.189	2.584	0.208	0.903	1.015	0.329	1.041	-0.614	0.171	-6.094	0.387	146
Item 68	0.034	0.312	4.533	0.177	2.213	0.205	0.897	1.031	0.355	1.071	-0.721	0.352	-6.226	0.773	129
Item 69	0.03	0.327	4.665	0.186	2.171	0.22	0.838	0.942	0.242	0.916	-1.107	-0.592	-7.535	-0.886	108
Item 70	0.019	0.238	5.221	0.232	2.698	0.263	0.976	1.134	0.328	1.178	-0.064	0.96	-4.711	1.247	90
Item 71	0.018	0.255	5.277	0.237	2.443	0.282	0.875	0.991	0.3	1.039	-0.587	-0.032	-4.867	0.329	69
Item 72	0.009	0.186	6.038	0.328	3.28	0.365	0.961	1.063	0.211	1.047	-0.04	0.345	-4.047	0.258	58

Note. Classical difficulty (p) represents the proportion of correct responses, and corrected item—total r represents the correlation between each item and the total score excluding that item. Items 1–9 were excluded from Rasch calibration because of pronounced ceiling effects and insufficient response variability; therefore, Rasch statistics are not reported for these items. In the primary Rasch analysis, post-discontinuation nonadministered items were coded as incorrect (0), whereas in the sensitivity analysis, the four observed incorrect responses triggering discontinuation were retained as incorrect and subsequent nonadministered items were treated as missing. *Administered, n* represents the number of participants who actually received each item. Infit and Outfit statistics are reported as mean-square (MnSq) and standardized t values.
